# Pulmonary Embolism and Coexisting Deep Vein Thrombosis: A Detrimental Association?

**DOI:** 10.3390/jcm8060899

**Published:** 2019-06-23

**Authors:** Elena-Mihaela Cordeanu, Hélène Lambach, Marie Heitz, Julie Di Cesare, Corina Mirea, Alix-Marie Faller, Anne-Cécile Cavaro, Anne-Sophie Frantz, Sebastien Gaertner, Valérie Schini-Kerth, Dominique Stephan

**Affiliations:** 1 Department of Hypertension, Vascular Disease and Clinical Pharmacology, Strasbourg Regional University Hospital, 67091 Strasbourg, France; helene.lambach@chru-strasbourg.fr (H.L.); marie.heitz2@chru-strasbourg.fr (M.H.); juliedicesare@hotmail.fr (J.D.C.); corina.mirea@chru-strasbourg.fr (C.M.); anne-cecile.cavaro@chru-strasbourg.fr (A.-C.C.); anne-sophie.frantz@chru-strasbourg.fr (A.-S.F.); sebastien.gaertner@chru-strasbourg.fr (S.G.); dominique.stephan@chru-strasbourg.fr (D.S.); 2 UMR 1260 INSERM Nanomédecie Régénérative, Faculté de Pharmacie, Université de Strasbourg, 67401 Illkirch, France; valerie.schini-kerth@unistra.fr; 3 Department of vascular surgery and vascular medicine, Reims University Hospital, 51100 Reims, France; alixfaller@hotmail.com

**Keywords:** venous thromboembolism, deep vein thrombosis, pulmonary embolism, severity

## Abstract

Background: The prognostic significance of coexisting deep vein thrombosis (DVT) in acute pulmonary embolism (PE) is controversial. This study aimed to provide routine patient care data on the impact of this association on PE severity and 3-month outcomes in a population presenting with symptomatic venous thromboembolism (VTE) from the REMOTEV registry. Methods and Results: REMOTEV is a prospective, non-interventional study of patients with acute symptomatic VTE, treated with direct oral anticoagulants (DOACs) or standard anticoagulation (vitamin K antagonists (VKA) or parenteral heparin/fondaparinux alone) for at least 3 months. From 1 November 2013 to 28 February 2018, among 1241 consecutive patients included, 1192 had a follow-up of at least 3 months and, among them, 1037 had PE with (727) or without DVT (310). The median age was 69 (55–80, 25th–75th percentiles). Patients with PE-associated DVT had more severe forms of PE (*p* < 0.0001) and, when DVT was present, proximal location was significantly correlated to PE severity (*p* < 0.01). However, no difference in all-cause mortality rate (hazard ratio (HR) 1.36 (CI 95% 0.69–2.92)), nor in the composite criterion of all-cause mortality and recurrence rate (HR 1.56 (CI 95% 0.83–3.10)) was noted at 3 months of follow-up. Conclusion: In REMOTEV, coexisting DVT was associated with a higher severity of PE, with no impact on short-term prognosis.

## 1. Introduction

Pulmonary embolism (PE) is a frequent, potentially fatal condition, with a wide spectrum of clinical features and protean prognosis. If its discovery on autopsy series ranges from 7% to 30%, symptomatic PE occurs in 1/1000 persons per year [1]. PE is the third cause of cardiovascular death and its early mortality is directly related to initial severity. The early death rate ranges from 0.5% in hemodynamically stable patients to 35%–58% in patients presenting with cardiogenic shock [2,3]. In order to detect patients at higher risk of complication during the acute phase, the Pulmonary Embolism Severity Index (PESI) and its simplified version (sPESI) integrating age, comorbidities, blood pressure, heart rate, and oxygen saturation have been validated in the estimation of mortality risk at 30 days [4,5]. In 2014, the European Society of Cardiology (ESC) adopted a 3-level risk classification of PE; (a) high risk in case of hemodynamic instability, (b) intermediate risk in case of normotensive patients with PESI Class III–V or sPESI elevation further assessed into two categories—intermediate low or intermediate high risk—according to the right ventricular (RV) function evaluation, (c) low risk in case of normotensive patients with PESI Class I–II or sPESI score of 0 and the absence of biomarker elevation or right heart dysfunction [6].

PE and deep vein thrombosis (DVT) are two replicates of the same pathology, frequently associated. Though one third of clinically-overt DVTs are associated with a silent PE it has been shown that isolated DVT has lower complication rates than PE [7,8,9]. Historically, the reported rates of concomitant presence of DVT in PE are ranging from 10% to 93%, but most recent studies found coexisting DVT in 56% to 61% of the symptomatic PE cases [10,11,12]. Whether the association of DVT and PE has a poorer prognosis than isolated PE is not known. A literature analysis shows opposing data regarding the association of DVT and outcomes such as death and recurrence [2,13,14].

Our study aimed to assess the correlation between the presence of concomitant DVT and PE severity, as well as 3-month outcomes (recurrence, death) in patients with acute PE. Thus, we conducted an observational study on a prospective cohort of venous thromboembolism (VTE) (REMOTEV registry) in order to advance our understanding of short-term PE outcomes.

## 2. Experimental Section

### 2.1. Study Design and Patient Selection

REMOTEV is an ongoing observational, prospective registry enrolling all consecutive patients hospitalized in the Vascular Medicine Unit of Strasbourg University Hospital for acute DVT and/or PE [15,16,17,18,19]. In the present study, we analyzed the clinical characteristics, comorbidities, treatment, and events during the first 3 months after VTE diagnosis in PE patients. Patients were informed about the purpose of the registry and gave oral consent to their participation according to the requirements of the local Ethics Committee. Data were recorded in a computerized anonymized form.

### 2.2. Requirements for VTE Diagnosis

The imaging procedures to confirm PE and DVT were validated diagnostic tests [15,16,17]. The initial PE and/or DVT (index event) was recorded. PE was confirmed by either computed tomography (CT) pulmonary angiogram or ventilation perfusion lung scan. Patients with symptomatic PE were routinely screened for DVT. The presence of DVT was assessed by ultrasonography from inferior vena cava to calf veins in both lower limbs.

### 2.3. Baseline Variables

Age, sex, weight, height, and comorbidities were collected. Comorbidities included chronic kidney disease (CKD) and active cancer. CKD was evaluated according to the Cockcroft creatinine clearance. For patients whose body weight was unknown, the estimated glomerular filtration rate (eGFR) was calculated using the abbreviated Modification of Diet in Renal Disease (MDRD) equation. VTE was classified as provoked or unprovoked depending on the presence or absence of the following associated risk factors: recent surgery (<3 months), prolonged immobilization (more than 3 days), recent travel (for more than 6 h) occurring within 1 month before VTE, known active cancer, pregnancy or a postpartum setting, and estrogen therapy (oral contraception or hormone replacement therapy) [18]. Known active cancer was defined as a recently diagnosed cancer (<6 months), cancer under treatment, or metastatic cancer. Cardiac biomarkers (troponin I, brain natriuretic peptide) and right ventricle parameters, measured by CT-scan or ultra-sound, were recorded. PE severity was classified according to the 2014 ESC Guidelines for PE management based on the short-term mortality risk (i.e., low risk, intermediate low, intermediate high, and high risk).

### 2.4. Treatment Regimens

The type, dose, and duration of anticoagulant drug therapy were at the attending physician’s discretion and were based on current recommendations [6,20,21,22]. Anticoagulation treatment consisted of rivaroxaban (15 mg twice daily for 21 days, followed by 20 mg once a day), apixaban (10 mg twice daily for 7 days, followed by 5 mg twice daily), which were the only direct oral anticoagulants (DOACs) reimbursed by the health insurance system in France, unfractionated heparin (UFH), low molecular weight heparin (LMWH) or fondaparinux, overlapping with and followed by international normalized ratio (INR) titrated vitamin K antagonists (VKA) or long-term parenteral anticoagulation including LMWH, or fondaparinux. Indications for thrombolysis, thromboaspiration, or surgical thrombectomy or implantation of an inferior vena cava filter were based on the current guidelines [6,20,21].

### 2.5. Follow-Up and Outcome Assessment

For the purpose of this study, the observation period ended 3 months from the date of the index event. All patient data were collected at the initial visit and then via phone interview at 1 month (+/−5 days) and 3 months (+/−10 days). The main evaluation criteria were: recurrent VTE, all-cause death, major adverse cardiovascular events (MACE), and major or non-major clinically relevant bleeding. Major and non-major clinically relevant bleeding events were classified according to the International Society on Thrombosis and Haemostasis (ISTH) criteria [23]. Recurrent VTE was considered as the cause of death when other causes were excluded and when PE could not be ruled out. Death was classified as due to PE, bleeding, cancer, cardiovascular causes (myocardial infarction, stroke, sudden death, and heart failure), or other. The evaluation criteria were adjudicated by senior physicians of the vascular medicine unit.

### 2.6. Statistical Analysis

This was a prospective observational study and therefore no formal power calculation was performed. Continuous variables were expressed as mean standard deviation (SD) or median with interquartile range (IQR) depending on the distribution. The normality of the distribution was assessed graphically and using the Shapiro–Wilk test. Categorical variables were presented as numbers of cases (percentages). Patients were divided according to the presence of concomitant DVT (PE with or without DVT) as well as PE severity (low risk versus intermediate/high risk PE). The association between a PE of intermediate or high risk and several baseline characteristics known to be correlated to PE severity was assessed by univariate analysis. Risk factors associated with PE severity having a univariate test considered significant were selected as a candidate for the multivariate logistic regression analysis. We compared the risk of death, major and clinically relevant non-major (CRNM) bleeding, or VTE recurrence between the two groups (PE with DVT versus PE without DVT). A composite factor including the three events was also created. Results were expressed as hazard ratio (HRs) with 95% confidence intervals (CI). A *p*-value < 0.05 was considered as statistically significant. The Kaplan–Meier estimator was employed to compute survival curves over the 3-month follow-up (FU). All analyses were performed using R software version 3.2.2 (www.r-project.org).

## 3. Results

### 3.1. Baseline Characteristics and Deep Vein Thrombosis Prevalence

**Figure 1:**
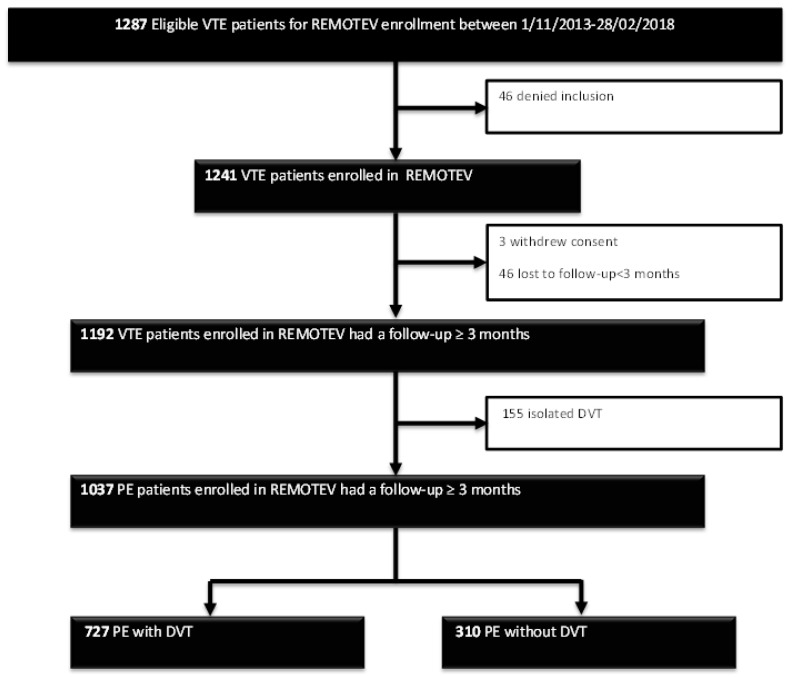
Study flowchart. DVT: deep vein thrombosis; PE: pulmonary embolism; VTE: venous thromboembolism. PE: pulmonary embolism; REMOTEV: Observational Prospective Monocentric Registry of Patients Suffering From Venous Thromboembolism.

Between 1 November 2013 and 28 February 2018, 1241 patients with VTE were included in the REMOTEV registry and 1192 had a follow-up of at least 3 months, as shown in Figure 1.

Among them, 1037 had PE with (70.1%) or without DVT (29.9%). There were 53% of women and the median age was 69 (55–80, 25th–75th percentiles). For 59% of the patients, the index event was considered unprovoked while 11% had a known active cancer. Anticoagulant treatment consisted of a direct oral anticoagulant (DOAC) in 73% of the cohort. Median hospital length of stay was 6 days (5–9, 25th–75th percentiles). The recommended duration of treatment was 3 months for 9% of patients, 6 months for 46% of patients, and without scheduled stop date for 45% patients. The baseline characteristics of all PE patients are summarized in Table 1 according to the presence of concomitant DVT or not. Our further analysis focused on coexisting DVT. Comparing DVT patients with DVT-free patients yielded several differences between groups as shown in Table 1.

### 3.2. DVT and PE Severity Assessment

**Figure 2:**
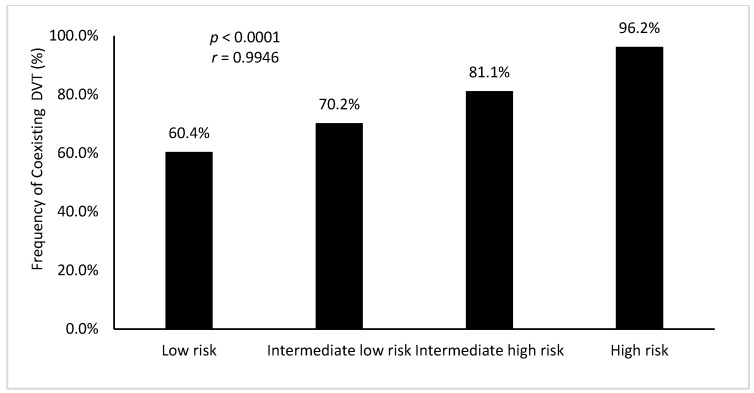
Presence of coexisting deep vein thrombosis (%) according to pulmonary embolism severity. DVT: deep vein thrombosis. *r*: Pearson’s correlation coefficient.

When DVT was present, it was proximal in 62% of cases. PE was at low risk for 35.1% of the patients, intermediate low for 38.3%, intermediate high for 24%, and high for 2.6% of the cohort. PE severity (intermediate or high risk) was strongly associated to the presence of DVT (*p* < 0.0001), in a linear manner, as shown in Figure 2. Furthermore, proximal location of DVT was significantly correlated with intermediate/high risk of PE (*p* < 0.01).

### 3.3. Risk Factors Associated with PE Severity

Univariate and multivariate analyses of factors associated to PE severity are presented in Table 2. According to univariate analysis, PE severity (low risk versus intermediate/high risk) was correlated with cancer, age, DVT, female sex, high blood pressure, diabetes, chronic obstructive pulmonary disease, renal impairment, and ischemic heart disease. As such, a multivariate Cox proportional hazards regression was performed to adjust for those covariates considered significant (*p* < 0.01 for univariate analysis) and found a strong correlation with cancer, age, DVT, and poor renal function, as shown in Table 2.

### 3.4. Concomitant DVT and Risk of Poor Outcomes after a 3-Month Follow-Up

Overall, 50 deaths, 21 thromboembolic recurrences (20 patients), 36 major bleedings (34 patients), and 63 CRNM bleedings (59 patients), were recorded over the 3-month FU. Although there were higher mortality and VTE recurrence rates in patients with DVT, statistical significance was not reached, as shown in Table 3.

**Figure 3:**
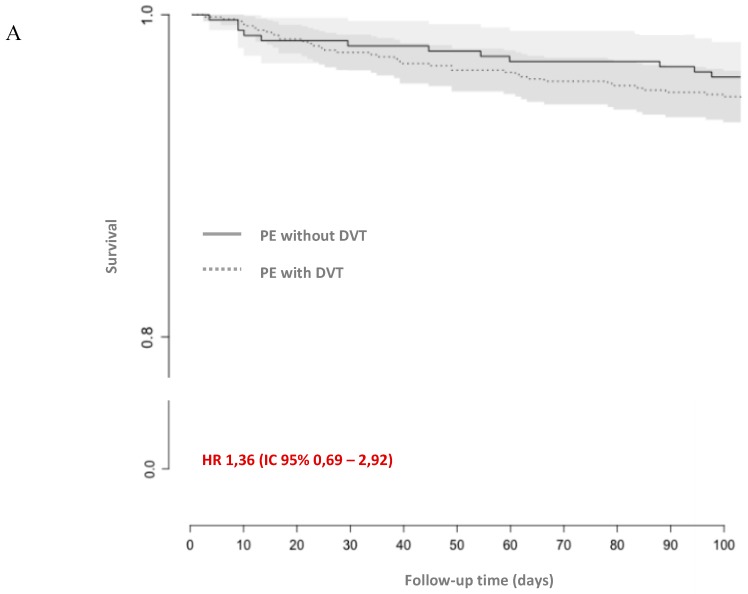

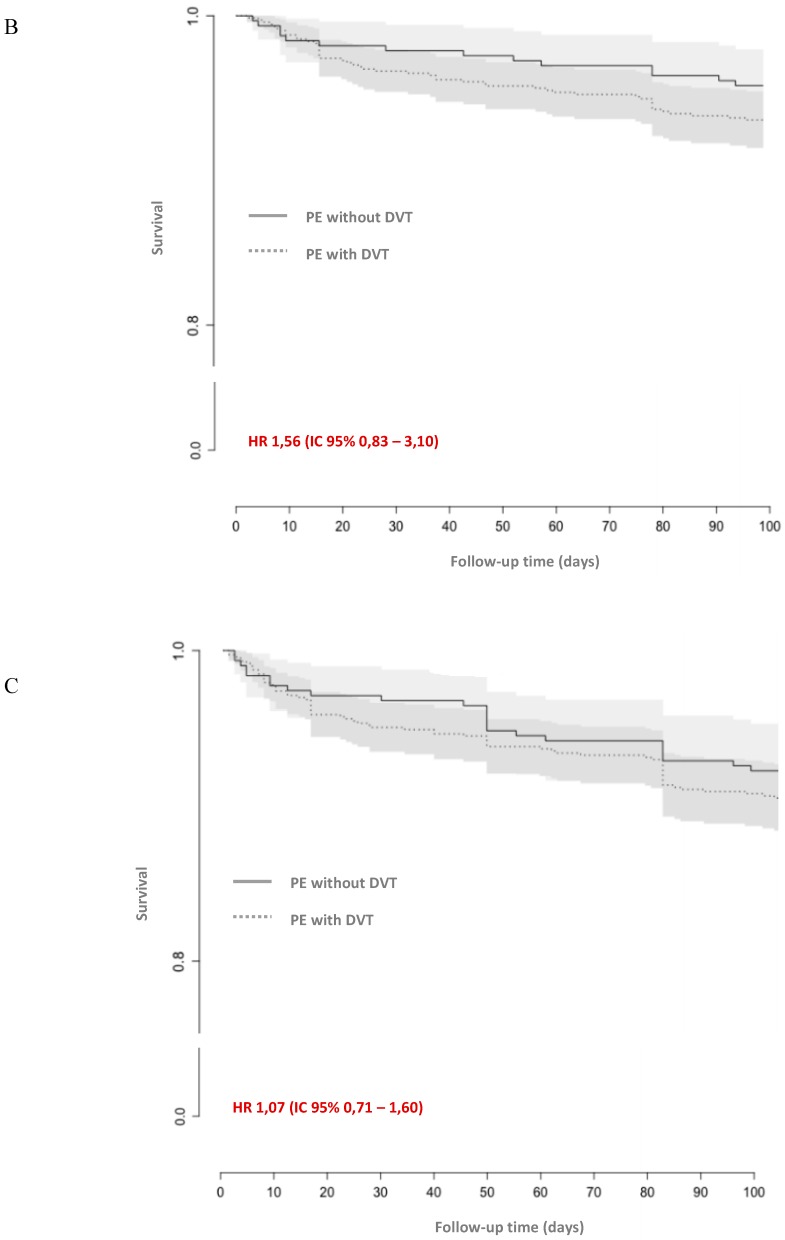
Kaplan–Meier curves of (**A**) all-cause mortality, (**B**) the composite of venous thromboembolism recurrence and all-cause mortality, (**C**) the composite of major and non-major clinically relevant bleeding, venous thromboembolism recurrence, and all-cause death. CI: confidence interval; HR: hazard ratio; DVT: deep vein thrombosis; PE: pulmonary embolism.

The composite criterion of VTE recurrence and all-cause mortality was more frequent in patients with DVT than without DVT, but the difference was not statistically significant; HR 1.56 (CI 95% 0.83–3.10), as shown in Figure 3.

## 4. Discussion

This was a prospective cohort study assessing PE severity and 3-month outcomes according to the presence of coexisting DVT in a consecutively recruited PE-population from the REMOTEV registry. Our study showed that, in a real-life setting, the presence of concomitant DVT was significantly associated with more severe forms of PE (intermediate or high risk). Furthermore, when a DVT was present, proximal location was significantly correlated to PE severity. The secondary study outcome which was a composite of VTE recurrence and overall mortality at 3 months after PE diagnosis showed no difference with respect to the presence of DVT.

Several studies showed that short-term outcomes were correlated to PE severity at presentation [24]. As such, an accurate risk stratification is of paramount importance. We have already shown in a prior study that sPESI, the central piece of the ESC prognostic model, underestimated the severity of PE since one third of low risk PE as defined by a sPESI of 0 had positive cardiac biomarkers or RV dilation [16]. Thus, improving risk stratification may lead to more suitable initial management and better prognosis.

In our study, 14% of patients had a complicated course including VTE recurrence and major or non-major clinically relevant bleeding totalizing 163 adverse events during the 3-month FU. This is the largest prospective cohort analyzing the significance of coexisting DVT in the era of DOACs, but it failed to prove any correlation between DVT and PE short-term adverse outcomes.

To date, published studies have design and methodology discrepancies showing conflicting results. Certain authors have identified a close relationship between coexisting DVT and PE prognosis (all-cause mortality and/or PE-related complications) [2,10,25]. Indeed, ICOPER (International Cooperative Pulmonary Embolism Registry) included 2442 patients with PE diagnosed on necropsy, lung scan, or pulmonary angiography and showed that DVT presence was inversely correlated to mortality risk (HR 0.5 (CI 95% 0.4–0.6)) [2]. Conversely, Jimenez et al. used a subgroup of the RIETE (The Computerized Registry of Patients with Venous Thromboembolism) population including 4476 patients for a validation cohort showing a 1.7-fold risk for all-cause death in patients with concomitant DVT, but their analysis had a retrospective design. In a meta-analysis of 10 cohorts (8859 patients), Becattini et al. found that the presence of a coexisting DVT was associated with higher PE severity and a 90% increase in the risk of 30-day all-cause mortality. However, 90-day PE-related adverse outcomes (VTE recurrence, PE-related death) were not significantly enhanced. Nonetheless, this meta-analysis included 10 cohorts of different methodology out of which only 2 studies totalizing 1003 patients had a prospective follow-up of at least 3 months [13,25].

Two other studies were published since this meta-analysis [26,27]. Lee et al. analyzed 141 PE patients but failed to establish any clinical significance of concomitant DVT for PE-related unfavorable outcomes and all-cause mortality [27]. More recently, Quezada et al. suggested in an analysis based on the PROTECT study including 848 patients that adding DVT testing to the ESC intermediate risk subpopulation improved the identification of patients with higher risk of complication [26].

One of the strengths of our report is to have systematically and thoroughly searched for DVT in the presence of PE which is not always performed in clinical practice when patients are asymptomatic although more than half of the DVTs associated to PE are known to be asymptomatic [28]. We have thus identified a high proportion of patients with DVT among PE patients (71%) that was similar to the one found by Hirmerova et al. and closer to reported rates of venographically-detected DVT (82%) than ultrasound-detectable ones (45%–55%) [25,28]. Distal DVT was present in one third of our population which is similar to the rates found by Hirmerova et al. and is in line with the lower risk of PE associated with distal DVTs [28]. The prospective design and high recruitment rate of our study account for some of its other strengths. However, this study has some limitations related to its monocentric design and, as like most registries, suffers from biases related to the absence of randomization.

In conclusion, our results show a strong correlation between DVT and PE severity as defined by the 2014 ESC model. We therefore believe that including DVT in PE severity assessment might improve risk stratification quality and pertinence with a direct therapeutic incidence. Thus, patients at higher risk of complication should benefit from more comprehensive in-hospital surveillance and more intensive therapy.

## Figures and Tables

**Table 1:** Demographic and clinical characteristics of the study population at baseline.

	Total *N* (%)/M ± SD/ M (Q1–Q3)	PE with DVT *N* (%)/M ± SD/ M (Q1–Q3)	PE without DVT *N* (%)/M ± SD/ M (Q1–Q3)	*p*-Value
*N*	1037	727 (70.1)	310 (29.9)	
Age (years)	69 (55–80)	71 (57–80)	67 (50–78)	0.002
Age ≥ 70 years old	499 (48.1)	368 (50.6)	131 (42.3)	0.008
Male	493 (47.5)	361 (49.7)	132 (42.6)	0.04
Weight (kg) *N* = 1018	77.5 (67–188)	78 (67–167)	77 (68–188)	0.47
Weight ≤ 50 kg	31 (3)	18 (2.5)	13 (4.3)	0.19
BMI (kg/m^2^) *N* = 987	27 (24–31)	27 (24–31)	27 (24–31)	0.91
BMI ≥ 30 kg/m^2^	324 (32.8)	230	94	0.69
eGFR (mL/min/1.73 m^2^) on admission	85.5 (67.2–104.3)	83 (65.1–100.7)	91.9 (70.8–112.2)	0.0001
eGFR ≥ 90	460 (44.4)	294 (40.4)	166 (53.7)	
60 ≤ eGFR < 90	381 (37.8)	285 (39.2)	96 (31.1)	
30 ≤ eGFR < 60	166 (16)	125 (17.2)	41 (13.3)	
eGFR < 30	29 (2.8)	23 (3.2)	6 (1.9)	
CrCl Cockcroft (mL/min) on admission	85.2 (58.7–119.6)	81.7 (55.5–114.1)	90.9 (64.7–128.4)	0.001
CrCl < 50 mL/min	177 (17.4)	137 (19.2)	40 (13.2)	0.02
Cardiovascular risk factors				
Hypertension	563 (54.3)	418 (57.5)	145 (46.8)	0.001
Diabetes	165 (15.9)	115 (15.8)	50 (16.1)	0.97
Dyslipidemia	344 (33.2)	251 (34.5)	93 (30)	0.17
Smoking (history or current)	417 (41.2)	287 (40.3)	130 (43.3)	0.40
Medical history				
Previous thromboembolism	332 (32)	230 (31.6)	102 (33)	0.71
PAD	32 (3.1)	20 (2.8)	12 (3.9)	0.71
CAD	62 (5.9)	41 (5.6)	21 (6.8)	0.57
COPD	56 (5.4)	32 (4.4)	24 (7.7)	0.04
Active cancer. total	89 (8.6)	68 (9.4)	21 (6.8)	0.21
Known thrombophilia	41 (3.9)	24 (3.3)	17 (5.5)	0.13
Antithrombotic treatment on admission				
Antiplatelet	202 (19.6)	131 (18.1)	71 (22.9)	0.08
Anticoagulation	66 (6.4)	40 (5.5)	26 (8.4)	0.11
PE severity				
Low risk	364 (35.1)	220 (30.3)	144 (46.5)	<0.0001
Intermediate low	397 (38.3)	279 (38.4)	118 (38.1)	
Intermediate high	249 (24)	202 (27.8)	47 (15.1)	
High risk	27 (2.6)	26 (3.5)	1 (0.3)	
DVT location				
Lower limbs	717 (69.1)	717 (98.6)	-	
Bilateral	127 (12.2)	127 (17.5)	-	
Proximal	454 (43.8)	454 (62.4)	-	
Distal	263 (25.3)	263 (36.2)	-	
Unusual site	25 (2.4)	25 (3.4)	-	
Isolated	10 (1)	10 (1.4)	-	
Type of VTE				
Unprovoked	613 (59.1)	430 (59.1)	183 (59)	1
IVC filter	13 (1.2)	12 (1.6)	1 (0.3)	0.12
PE Thrombolysis/-ectomy/-aspiration	18 (1.7)	17 (2.4)	1 (0.3)	0.02
Anticoagulant treatment at discharge				
DOAC	756 (72.9)	528 (72.6)	228 (73.5)	0.81
VKA	135 (13)	92 (12.6)	43 (13.9)	0.66
LMWH/Fondaparinux	145 (14)	106 (14.6)	39 (12.6)	0.44
No anticoagulant	1 (0.1)	1 (1.4)	0	1
Anticoagulant treatment duration				
3 months	93 (8.9)	53 (7.2)	40 (12.9)	0.007
6 months	474 (45.7)	342 (47)	132 (42.6)	0.21
Indefinite	469 (45.2)	331 (45.6)	138 (44.5)	0.81

BMI: body mass index; CAD: coronary artery disease; COPD: chronic obstructive pulmonary disease; eGFR: estimated glomerular filtration rate; DVT: deep venous thrombosis; IVC: inferior vena cava; LMWH: low molecular weight heparin; PAD: peripheral artery disease; PE: pulmonary embolism; SD: standard deviation; VKA: vitamin K antagonists; VTE: venous thromboembolism; DOAC: direct oral anticoagulants.

**Table 2:** Univariate and multivariate analysis of baseline risk factors for pulmonary embolism severity.

Risk Factor	Unadjusted HR (95% CI)	*p*–Value	Adjusted HR (95% CI)	*p*–Value
Age > 70 years old	7.02 (5.15–9.65)	<0.001	4.85 (3.46–6.87)	<0.001
Female sex	1.47 (1.13–1.92)	<0.01	1.41 (1.04–1.91)	<0.05
High blood pressure	3.42 (2.60–4.52)	<0.001	1.56 (1.12–2.17)	<0.01
Diabetes	1.84 (1.24–2.77)	<0.01	0.95(0.60–1.52)	NS
eGFR < 60 mL/min/1.73 m^2^	5.35 (3.34–8.95)	<0.001	2.70 (1.64–4.61)	<0.001
Cancer	26.82 (7.12–225.30)	<0.001	30.64 (9.30–189.37)	<0.001
COPD	3.41 (1.57–8.45)	<0.001	4.00 (1.82–9.83)	<0.01
Ischemic heart disease	3.36 (1.62–7.86)	<0.001	1.50 (0.69–3.56)	NS
Presence of DVT	1.99 (1.50–2.65)	<0.001	2.03 (1.47–2.82)	<0.001
Known thrombophilia	0.36 (0.18–0.72)	<0.01	0.88 (0.42–1.77)	NS

CI: confidence interval; DVT: deep vein thrombosis; eGFR: estimated glomerular filtration rate; HR: hazard ratio; NS: not significant.

**Table 3:** Three-month adverse outcomes according to the presence of coexisting deep vein thrombosis.

Outcome	Total *n* = 1037	DVT *n* = 727	DVT-Free *n* = 310	HR (CI 95%)	*p*-Value
All-cause death	50	38	12	1.36 (0.69–2.92)	0.42
VTE recurrence	21	15	6	1.28 (0.44–4.55)	0.80
Major bleeding	34	23	11	0.88 (0.40–20.4)	0.70
Clinically relevant non-major bleeding	59	40	19	0.89 (0.49–1.65)	0.66

CI: confidence interval; DVT: deep vein thrombosis; HR: hazard ratio; n: number; VTE: venous thromboembolism.
